# Food support provision in COVID-19 times: Organizational data from Greater Manchester

**DOI:** 10.1016/j.dib.2022.107918

**Published:** 2022-02-04

**Authors:** Filippo Oncini

**Affiliations:** University of Manchester*,* Sustainable Consumption Institute, UK

**Keywords:** Food resources, Food aid, Welfare food scheme, Food waste recycling, Poverty, Greater Manchester, Food bank, COVID19

## Abstract

The dataset presented in this paper contains information on 55 food support providers active in Greater Manchester during the COVID-19 crisis. Survey data were collected in June 2020 to obtain standardized information on the obstacles, needs, and prospects of the food support providers of the region immediately after the first COVID-19 wave. Although the sample is mainly composed of food banks, it also includes other providers such as food pantries, food clubs and meal providers. The data allows to draw some preliminary conclusions on the emergency response put in place and to highlight the most common difficulties faced by the organizations. To this purpose, the dataset contains variables that capture information related (i) to the impact of COVID-19 on organizational procedures and management, and (ii) to the characteristics of different food support provider before the COVID-19 outbreak.

## Specifications Table

*Every section of this table is mandatory. Please enter information in the right-hand column and remove all the instructions in grey, italicised text.*

**Table:** 

Subject	Social Science – Social Science
Specific subject area	Organization studies
Type of data	Text, Table
How the data were acquired	Standardised questionnaire to food support providers based in Greater Manchester. The dataset was analysed looking at percentage frequency distributions of the following variables: type of food support provider, how often the organizations turned people away, changes in donations and nutritional value, reported shortages, number of weeks existing stock/reserves will last, impact of COVID-19 on financial stability, management, functioning, and social atmosphere.
Data format	Mixed (Raw and cleaned)
Description of data collection	Data comprise information on 55 organizations that provided food support in Greater Manchester during the COVID-19 crisis. The data were generated with a CAWI/CATI technique in June 2020 after identifying all the food support providers potentially active in the metropolitan county. The survey respondents are directors and spokespersons of organizations based in Greater Manchester.
Data source location	Institution: UK Data Service Reshare Repository, University of Essex, Colchester City/Town/Region: Essex Country: UK
Data accessibility	Repository name: UK Data Service ReShare Repository Data identification number: 10.5255/UKDA-SN-854874 Direct URL to data: https://reshare.ukdataservice.ac.uk/854874/ Data can be freely accessed at the link. Alternatively, the .csv file can be downloaded directly from the article.
Related research article	Oncini, F. (2021). Food support provision in COVID-19 times: a mixed method study based in Greater Manchester. Agriculture and human values, 38, 1201–1213. https://doi.org/10.1007/s10460-021-10212-2

## Value of the Data

• These data present information on several food support providers active in Greater Manchester during June 2020, particularly on the impact of COVID-19 and on the characteristics of the organizations before the virus outbreak.
• These data are a valuable source of information to understand how the charitable food provision sector reacted to the restriction measures adopted after the first COVID-19 wave and to investigate the operational characteristics of different providers.
• Data can be employed to further investigate the immediate impact of COVID-19 on food support providers. For instance, they allow to understand how food was delivered to users and whether certain types of food providers faced more difficulties during the crisis.
• Researchers can extend the study and replicate the survey to investigate the current impact of COVID-19 on food support providers. New data could be then compared with June 2020 data to grasp differences between short-term and long-term effects of COVID-19 on the charitable food provision sector.
• Researchers interested in food poverty and food charities can use the dataset to visualize descriptive statistics on the sector.
• The data could be also used to explore differences between organizations operating with distinct provision models (e.g. food banks vs pantries).
• The second part of the questionnaire could be used to replicate the survey in other regions or countries.

## 1 Data Description

*Dataset:* The dataset contains information on 55 food support providers active in June 2020 in Greater Manchester (UK). The dataset includes 83 variables related (i) to the COVID-19 crisis and (ii) to the characteristics of the organization before the pandemic.

The first set of variables gathers information on the impact of the pandemic on the operations of the organization (e.g. the number of volunteers and staff members, the volume of food and monetary donations, on the overall resilience and capacity to keep providing food support, and on the type of users served by the organization).

The second set of variables captures more generic information on the providers (e.g. religious affiliation, foundation, red-tape), as well as data on their organization before the virus outbreak (e.g. type of food offered, nutritional quality, food sources, number of users per week). Each variable has a unique ID and a label to ease the use of the dataset. The ID can be used to trace back the item in the questionnaire and in the codebook.

*Questionnaire:* The questionnaire file contains an introductory note with details on the data collection procedure and the full questionnaire used to gather the data (with consent forms). Some questions – in red in the text – signal the variables that were excluded from the dataset to protect the anonymity of the participants. All questions have a unique ID that links each item with the variables.

*Codebook:* The codebook file is a .txt file with detailed information and descriptive statistics for each variable. Each variable in the codebook has an ID that corresponds to the dataset and questionnaire ID, the variable label, the type of variable, the range, the unique values (used to determine whether a variable is categorical or continuous) and the number of missing values. In addition, the codebook shows, for categorical variables, the frequencies, the response categories and the numeric labels; for continuous variables, the mean and the standard deviation.

## 2 Experimental Design, Materials and Methods

Since the Great Recession (2007–2009), the number of food charities all over the UK has dramatically increased over time [1]. During the COVID-19 crisis, their role became even more crucial, as increases in the number of food parcels distributed were reported from several organizations [2,3]. Focusing on food support providers active in the Greater Manchester metropolitan county during the pandemic, this dataset collects information on the main challenges and the prospects of the organizations providing food aid to people in need. In addition, the dataset also contains information on the pre-COVID-19 operations and permits to explore differences and similarities between several providers.

### 2.1 Sample

The sample includes 55 food support providers active in the Greater Manchester area in June 2020. The sample corresponds to 50% of the population of the organizations providing food support that were able to remain open after the first COVID-19 wave. Data were collected throughout June 2020. First, the datasheet containing contact details for 222 services connected with food aid throughout Greater Manchester was extracted from the open-data map of food support providers created by Greater Manchester Poverty Action (GMPA), a charity that works to prevent and reduce poverty across Greater Manchester. Additional food support providers were retrieved on the Mutual Aid Groups Map (26), and by sending a link to the online questionnaire using the GMPA newsletter (9).

### 2.2 Data collection

The contact database was then shared with a research agency that administered the questionnaire via CATI (Computer-Assisted Telephone Interviewing) or CAWI (Computer-Assisted Web Interviewing) once operators had spoken to directors or spokespersons. The list of 257 food support providers contained 33 duplicates. Enquiries revealed that 41 did not provide food support or any type of aid; and 73 did not respond to several attempts to reach them, or their contact details were out of date. This latter group may have consisted of organizations that had to shut down for lack of volunteers or suitable spaces to reorganize support. Eventually, 55 directors/spokespersons participated in the survey and 55 either refused, or it was not possible to secure a CATI/CAWI interview (50% of the ‘active’ population).

## Ethics Statement

The research obtained ethical clearance from the University of Manchester Ethics Committee (2020-9377-15273).

## CRediT authorship contribution statement

**Filippo Oncini:** Conceptualization, Methodology, Data curation, Writing – original draft, Writing – review & editing, Project administration, Funding acquisition.

## Declaration of Competing Interest

The authors declare that they have no known competing financial interests or personal relationships that could have appeared to influence the work reported in this paper.

## Appendix Supplementary materials

Image, application 1

Image, application 2
