# Supplementary material for: Food support provision in COVID-19 times: Organizational data from Greater Manchester
Source: Data Brief. 2022 Feb 4;41:107918. doi: 10.1016/j.dib.2022.107918 (PMC8813765; doi:10.1016/j.dib.2022.107918)
Supplement: Supplementary file 1 [file mmc1.pdf]

Introductory notes to the survey

Survey data were collected to obtain standardized information on the characteristics of different food support providers active in Greater Manchester and on the impact of COVID-19 on their operations. First, the datasheet containing contact details for 222 services connected with food aid throughout Greater Manchester was extracted from the open-data map of food support providers created by Greater Manchester Poverty Action (GMPA). To this dataset details of 26 food support providers found on the Mutual Aid Groups Map, and 9 others were reached by sending a link to the questionnaire in the GMPA newsletter to get as close as possible to the statistical population. The contact database was then shared with a research agency that administered the questionnaire via CATI (Computer-Assisted Telephone Interviewing) or CAWI (Computer-Assisted Web Interviewing) once operators had spoken to directors or spokespersons.

The list of 257 food support providers contained 33 duplicates. Enquiries revealed that 41 did not provide food support or any type of aid; and 73 did not respond to several attempts to reach them, or their contact details were out of date. This latter group may have consisted of organizations that had to shut down for lack of volunteers or suitable spaces to reorganize support. Eventually, 55 directors/spokespersons participated in the survey and 55 either refused, or I could not secure a CATI/CAWI interview (50% of the 'active' population)

The dataset is available in dta format and response categories follow the numbering reported after each question below. Some questions – in red in the texts – were excluded from the dataset to protect the anonymity of the participants. Please contact Filippo.oncini@manchester.ac.uk for any further information you may need.

Food Support Providers Questionnaire - CATI

Good morning/afternoon. My name is ______________ and I am calling from Teamsearch on behalf of the University of Manchester. Could I please speak to a director or spokesperson of this food support provider?

ONCE CONNECTED:

ONCE CONNECTED My name is calling from Teamsearch on behalf of the University of Manchester. Recently, you should have received a participant information sheet with all the details regarding the study and the data collection procedure through the GMPA (Greater Manchester Poverty Action) newsletter. Did you have time to read the participant information sheet?

IF YES: Continue below

IF NO: Would you like me to send you again the Participant information sheet and reschedule the call?

As the email explained, we're currently conducting a survey to explore how different food support providers are organised, and how they are facing the crisis provoked by COVID-19. The questionnaire should take around 20 minutes to complete and there will be a £10 donation made to your organisation following completion of the questions. An additional £30 will be donated if you accept to participate to another interview with the principal investigator of the project. Would you have time for that now?

IF THE PERSON REFUSES: Would you be willing to respond to an online version of the questionnaire? You will still be entitled to receive the £10 donation.

IF NEEDED: In particular, the project aims to understand which strategies have been put in place by food support providers, what have been the main obstacles, how the spread of the virus has affected the everyday life of the organisation and what alternative solutions could be envisioned to tackle food insecurity in the future.

ONCE AGREED: Before we start, I would just like to confirm that I am calling from Teamsearch and we are an independent research company. Everything that you say to me will be treated in the strictest confidence in accordance with the Market Research Society guidelines, General Data Protection Regulation and the Data Protection Act.

All the information that we collect about the food support provider during the course of the call will be kept strictly confidential. You will not be able to be identified or identifiable in any reports or publications. Your food support provider will also not be identified or identifiable. Any data collected about you in the online questionnaire will be stored in the research data storage of the University of Manchester in a form protected by passwords and other relevant security processes and technologies. Data collected may be shared in an anonymised form to allow reuse by other third parties. These anonymised data will not allow any individuals or their institutions to be identified or identifiable.

Your participation in this study is completely voluntary. You have the right to withdraw from the study at any time without penalty. You are entitled to the £10 regardless of your decision

to answer any questions or to stop the interview. We will keep the information you give us confidential. Study files will be kept in a secure place.
There are no expected discomforts, disadvantages or risk to yourself by taking part in the research. Further to this is, you are not required to answer any questions that you do not wish to answer and you can retract from the research study at any time with no penalty or loss of benefits to which you are otherwise entitled.
Before you agree to participate in the study, make sure that you have the answers to any questions you might have. In case you would like to ask more questions about the project, please send an email to Dr Filippo Oncini at this address: Filippo.oncini@manchester.ac.uk and we can reschedule the call.

If you would like to participate to the study, I will need to record your verbal agreement to the following statements. Can you please agree to the following by stating yes or no.

1 Do you confirm that you have read the information sheet for the present study and have had the opportunity to consider the information and ask questions and had these answered satisfactorily?
2 Do you confirm you are 18 years old or older?
3 Do you confirm that you understand that participation in the study is voluntary and that you are free to withdraw at any time without giving a reason and without detriment to yourself and that you understand that it will not be possible to remove the data from the project once it has been anonymised and forms part of the data set?
4 Do you agree that any data collected may be published in anonymous form in academic books, reports or journals?
5 Do you agree to take part in this study?

Thank you, we can now start the questionnaire.

In this first section, I would like to ask your opinion on how COVID-19 has affected the organisation and how it operates.

Q1. What type of food support provider are you? [READ OUT]

1. Trussell Trust food bank
2. Independent food bank
3. Food Club
4. Food Pantry
5. Meal provider
6. Other (specify)
7. We are not a food support provider [CLOSE]

Q2. How many distribution points/centres do you have? _________

Q2b. Are you providing other types of support besides food provision? [MULTI] [READ OUT]

1. No, we just provide food items
2. Referral to other sources of financial support
3. Referral to advice services (e.g. debt, housing, welfare, employment advice)
4. Provision of non-food items (e.g. toiletries)
5. Issuing fuel vouchers
6. Delivering prescriptions
7. Providing phone top up
8. Befriending and/or check-up calls
9. Other (please state)
10. Don't know [DO NOT READ OUT]
11. Refused [DO NOT READ OUT]

Q3. On a scale from 1 to 5, where 1 is "Not at all" and 5 is "Very much so", to what extent would you say COVID-19 has affected the following: [READ OUT EACH STATEMENT IN TURN] [SINGLE]

	Not at All				Very much So
Your organisation overall	1	2	3	4	5
The financial stability of the food support provider	1	2	3	4	5
The social atmosphere of the food support provider	1	2	3	4	5
The management of the food support provider	1	2	3	4	5

The functioning of the food support provider	1	2	3	4	5

Q4. Thinking about the following aspects of your organisation, how have each of them changed since the beginning of the COVID-19 outbreak? [READ OUT SCALE AND EACH STATEMENT IN TURN] [SINGLE]

	Decreased	Stayed the Same	Increased
Number of volunteers	1	2	3
Number of paid staff working	1	2	3
Number of users	1	2	3
Total opening hours	1	2	3
Volume of food donations	1	2	3
Volume of monetary donations	1	2	3
Volume of food wasted	1	2	3
Nutritional value of the food	1	2	3

Q5. Before Covid-19 outbreak, how often did your organization serve the following types of users? [READ OUT SCALE AND EACH STATEMENT IN TURN] [SINGLE]

	Frequently	Occasionally	Rarely	Never
Asylum seekers	1	2	3	4
Homeless people	1	2	3	4
Families with children	1	2	3	4
Single adults	1	2	3	4
Couples without children	1	2	3	4
Elderlies	1	2	3	4

Q6. After Covid-19 outbreak, would you say that attendance of the following types of users has... [READ OUT SCALE AND EACH STATEMENT IN TURN] [SINGLE]

	Decreased	Stayed the same	Increased	Don't know
People seeking asylum	1	2	3	4
Homeless people	1	2	3	4
Families with children	1	2	3	4
Single adults	1	2	3	4
Couples without children	1	2	3	4
Elderly people	1	2	3	4

Q7. How does the organization usually deliver food to the users? [READ OUT] [SINGLE]

1. Home delivery
2. Attendance to the distribution centre
3. Both
4. Other (specify)

Q8. On a scale from 1 to 4, where 1 is "Frequently" 2 is "Occasionally", 3 is "Rarely" and 4 is "Never", during the last few weeks, how often have you needed to turn eligible people away [READ OUT EACH STATEMENT IN TURN] [SINGLE]:

	Frequently	Occasionally	Rarely	Never
Due to lack of food	1	2	3	4
Due to lack of staff capacity	1	2	3	4
Due to lack of volunteer capacity	1	2	3	4
Due to the lack of a valid food voucher	1	2	3	4
For other reasons (specify)	1	2	3	4

Q9. Roughly, how much food, in terms of meals/equivalent, has the organization been giving out in a usual week since the beginning of the emergency? [OPEN] __________

Q10. Roughly, how many total weekly visits did you see in the last month? [OPEN] __________

Q11. Roughly, how many weeks will your existing food stocks last at current levels of demand? [OPEN] __________

1. This is not a problem for the organization
2. Don't know [DO NOT READ OUT]

Q12. Roughly, how many weeks will your existing cash reserves last at current levels of demand? [OPEN] __________
1. This is not a problem for the organization
2. Don't know [DO NOT READ OUT]

Q13. Do you have facilities for distributing chilled food? [SINGLE]
1. Yes
2. No
3. Don't know [DO NOT READ OUT]
4. Refused [DO NOT READ OUT]

Q14. During the last few weeks, have you needed to refuse chilled food donation because of lack of facilities? [SINGLE]
1. Yes
2. No
3. Don't know [DO NOT READ OUT]
4. Refused [DO NOT READ OUT]

Q15. Do you have facilities for distributing frozen food? [SINGLE]
1. Yes
2. No
3. Don't know [DO NOT READ OUT]
4. Refused [DO NOT READ OUT]

Q16. During the last few weeks, have you needed to refuse frozen food donation because of lack of facilities? [SINGLE]
1. Yes
2. No
3. Don't know [DO NOT READ OUT]
4. Refused [DO NOT READ OUT]

Q17. On a scale from 1 to 5, where 1 is "Not at all" and 5 is "Very much so", how much is the organization short on food? [READ OUT] [SINGLE]:

Not at All				Very much So
1	2	3	4	5

Q18. If so, what food items do you need most? [READ OUT] [OPEN]:

____
____
____

Q19. On a scale from 1 to 5, where 1 is "Not at all" and 5 is "Very much so", how much is the organization short on volunteers? [READ OUT] [SINGLE]:

Not at All				Very much So
1	2	3	4	5

Q20. On a scale from 1 to 5, where 1 is "Not at all" and 5 is "Very much so", how much is the organization short on staff members? [READ OUT] [SINGLE]:

Not at All				Very much So
1	2	3	4	5

Q21. Could you briefly tell me in your own words what have been the major changes in the overall organization after Covid-19 outbreak? [READ OUT] [OPEN]:

____
____
____

Q22. Could you briefly tell me, in your own words, what are the immediate needs of your food support provider? [READ OUT] [OPEN]:

____
____
____

Q23. Overall, how resilient do you feel your organisation is likely to be against the COVID-19 crisis? [READ OUT] [SINGLE]

1. Not at all resilient
2. Not very resilient
3. Fairly resilient
4. Very resilient
5. Don't know [DO NOT READ OUT]
6. Refused [DO NOT READ OUT]

I now would like to ask you some questions regarding the organisation of the food support provider. Please answer the following questions according to how your organisation normally operates, i.e. how it operated before the onset of COVID-19.

Q24. When was your organization founded? [READ OUT IF NEEDED] [OPEN] __________ [add counter with years 1900, 1901…]

1. Don't know [DO NOT READ OUT]
2. We have founded the organization during the Covid-19 emergency

Q25. And for how long have you been offering food support? [MUST BE LESS THAN Q1] [READ OUT IF NEEDED] [OPEN] __________ [add counter with years number 1 2 3 4…]

1. Don't know [DO NOT READ OUT]
2. Since the foundation of the organization
3. We have started offering food during the Covid-19 emergency

Q26. Is your organisation faith based or secular? [READ OUT IF NEEDED] [SINGLE]

1. Faith-based
2. Secular
3. Mixed
4. Don't know [DO NOT READ OUT]

[IF A OR C AT Q26]

Q27. Which faith(s) is your organisation affiliated with? [READ OUT IF NEEDED] [MULTI]

1. Christianity
2. Islam
3. Hinduism
4. Buddhism
5. Judaism
6. Other (specify)

Q28. How often do you generally offer the following? [READ SCALE AND EACH STATEMENT IN TURN] [SINGLE]

	Frequently	Occasionally	Rarely	Never
Warm Meals	1	2	3	4
Tinned Food	1	2	3	4
Packaged Meals (eg. microwave meals)	1	2	3	4

Cereal	1	2	3	4
Fresh Fruit	1	2	3	4
Fresh Vegetables	1	2	3	4
Fresh Meat	1	2	3	4
Fresh Cheese	1	2	3	4
Pasta	1	2	3	4
Bread	1	2	3	4
Salty snacks (e.g. crisps)	1	2	3	4
Sweets	1	2	3	4
Frozen food	1	2	3	4
Chilled food	1	2	3	4
Household items	1	2	3	4

Q29. How would you describe the nutritional quality of the food your organisation offers? [READ OUT SCALE] [SINGLE]

Inadequate				Excellent
1	2	3	4	5

Q30. How often does your organisation receive food from the following sources? [READ SCALE AND EACH STATEMENT IN TURN] [SINGLE]

	Frequently	Occasionally	Rarely	Never
Donations from individuals	1	2	3	4
Direct purchasing	1	2	3	4
Donations of surplus directly from retailers, manufacturers, or producers	1	2	3	4
Donations of surplus indirectly from retailers, manufacturers, or producers, e.g. through Fareshare	1	2	3	4

Other (specify)	1	2	3	4

Q31. Roughly, how many total weekly visits do you typically see in each season [OPEN]?

1. Winter _____
2. Spring _____
3. Summer _____
4. Autumn _____

Q32. If red tape is defined as burdensome administrative rules and procedures that have negative effects on the organisation's effectiveness, how would you assess the level of red tape in your organisation? [READ OUT SCALE] [SINGLE]

No red tape — Excessive red tape

0	1	2	3	4	5	6	7	8	9	10

Q33. What criteria do you use to determine eligibility for your support [READ OUT]?

1. No criteria
2. Limited referral from other agencies
3. Unlimited referral from other agencies
4. Other criteria (please specify)

Q34. How many of each of the following types of worker are registered to work at your organisation [OPEN]?

1. Volunteers: ___
2. Paid workers: ___

Q35. Roughly how much funding do you receive from each of the following channels per year [OPEN]?

1. Public sector: £_______________
   i. Don't know [DO NOT READ OUT]
   ii. Refused [DO NOT READ OUT]
2. VCSE sector £_______________
   i. Don't know [DO NOT READ OUT]
   ii. Refused [DO NOT READ OUT]
3. Private sector: £_______________
   i. Don't know [DO NOT READ OUT]
   ii. Refused [DO NOT READ OUT]
4. Individual donations £_______________

i. Don't know [DO NOT READ OUT]
ii. Refused [DO NOT READ OUT]

Q36. Roughly how much are your organisation's total annual outgoings [OPEN]?

1. £__________________
2. Don't know [DO NOT READ OUT]
3. Refused [DO NOT READ OUT]

Q37. How well do you feel your organisation was generally able to cover its costs? [READ OUT] [OPEN]

1. We constantly struggled to cover our costs
2. We sometimes struggled to cover our costs
3. We rarely struggled to cover our costs
4. We never struggled to cover our costs

C1. Thank you. That is the end of the questionnaire. We'd like to finally ask if you'd be willing to take part in an online interview with Dr Filippo Oncini, a researcher based at the University of Manchester, for a more in-depth discussion on how your organisation is being affected by COVID-19. The discussion will last approximately 1 hour via the free video conferencing app (e.g. Zoom, Skype), at a time and date convenient to you, and as a thank you for taking part, we will be donating an additional £30 to your organisation. Would you be willing to participate in this?

a. Yes (record details as below)
    i. Name: _________________________
    ii. Mobile number: _______________________
    iii. Email: ________________________
    iv. Preferred time/date: ____________________
b. No

Finally, would you like to receive an update on the research findings when these will be available?

a. Yes (record email below)
    i. Email: ________________________

Those are all the questions that I need to ask you, all that remains is to thank you for your time. Enjoy the rest of your day. Would you like our telephone number to check our credibility or make comments regarding this interview? (Teamsearch - 01422 360 371) You can also find us on the MRS online registry. To find this just Google 'MRS members list' and it is the first link returned at the top of the Google page. Would you like to know more about the study? Please get in touch with Dr Filippo Oncini via email at the following address: filippo.oncini@manchester.ac.uk "
